# Cross-Language Speech Emotion Recognition Using Bag-of-Word Representations, Domain Adaptation, and Data Augmentation

**DOI:** 10.3390/s22176445

**Published:** 2022-08-26

**Authors:** Shruti Kshirsagar, Tiago H. Falk

**Affiliations:** Institut National de la Recherche Scientifique, University of Quebec, Montréal, QC H3C 5J9, Canada

**Keywords:** cross-language speech emotion recognition, bag of audio words, modulation spectrum, domain adaptation, data augmentation

## Abstract

To date, several methods have been explored for the challenging task of cross-language speech emotion recognition, including the bag-of-words (BoW) methodology for feature processing, domain adaptation for feature distribution “normalization”, and data augmentation to make machine learning algorithms more robust across testing conditions. Their combined use, however, has yet to be explored. In this paper, we aim to fill this gap and compare the benefits achieved by combining different domain adaptation strategies with the BoW method, as well as with data augmentation. Moreover, while domain adaptation strategies, such as the correlation alignment (CORAL) method, require knowledge of the test data language, we propose a variant that we term N-CORAL, in which test languages (in our case, Chinese) are mapped to a common distribution in an unsupervised manner. Experiments with German, French, and Hungarian language datasets were performed, and the proposed N-CORAL method, combined with BoW and data augmentation, was shown to achieve the best arousal and valence prediction accuracy, highlighting the usefulness of the proposed method for “in the wild” speech emotion recognition. In fact, N-CORAL combined with BoW was shown to provide robustness across languages, whereas data augmentation provided additional robustness against cross-corpus nuance factors.

## 1. Introduction

Speech emotion recognition (SER) is an emerging field in affective computing that, as the name suggests, has as its goal the detection or characterization of a speaker’s emotional states based on analysis of the speech signal alone. SER can have applications across a wide range of domains, from call centers, to smart cars, healthcare, and education, to name a few. Emotion-aware human–machine interfaces are starting to emerge in the market via start-ups, such as audEERING, Nemesysco, Nexidia, and Emospeech. “In the wild” SER, however, is still very challenging as there are a number of parameters that can vary between training and testing conditions, including but not limited to the types of emotions collected, labeling schemes, sampling rates, environmental conditions, microphone settings, and speakers, as well as spoken languages and cultural background, just to name a few. These cross-corpus changes are known to severely hamper SER performance [1,2,3,4].

While many studies have explored the issue of cross-corpus SER (e.g., [1,2,5,6,7]), in this paper, we focus on the mismatch due to different languages. Commonly, cross-lingual emotion prediction has relied on three methods: feature normalization [3,6,8,9,10], domain adaptation (DA) [8,11,12], or transfer learning [2,4,13,14]. We pay particular attention to domain adaptation methods, which have seen great success in computer vision tasks (e.g., [15]) but are still under-explored in SER tasks. Domain adaptation improves the generalization of the SER system by minimizing the distribution shift between the source (training) and target (testing) data, including shifts due to varying languages. DA separates the two domains via measures of maximum mean discrepancy, correlation distances, or even by creating a shared representation of the source and target data [16].

Alternately, bag-of-word (BoW) [17] and data augmentation methodologies [18,19] have also been explored as ways to remove cross-corpus biases. BoW has been used for text-based sentiment analysis, as well as multimodal emotion recognition systems [20,21], and was recently explored for cross-lingual SER tasks [17,19]. Data augmentation, in turn, has been shown to provide some robustness against cross-corpus mismatches, including cross-language mismatches [18]. While DA, BoW, and data augmentation have been explored individually for cross-language SER tasks in the past, their combinations have yet to be explored. This paper aims to fill this gap, and experiments with SER tasks in German, Hungarian, Chinese, and French are performed. In particular, in this paper, the following contributions are made:

1. We explore the combination of DA and BoW for improved cross-language SER. Experiments with the BoW methodology before or after domain adaptation are performed to assess their advantages/disadvantages. Different DA methods are explored to gauge their effects on overall cross-language SER. In particular, the CORAL [22], and Subspace alignment-based domain adaptation (SA-DA) are compared.
2. A variant of the CORAL method is proposed for cross-language SER. The method, termed N-CORAL, makes use of a third unseen unlabeled dataset/language to adapt both domain and source data, in essence normalizing both training and test datasets to a common distribution, as typically done with domain generalization.
3. Lastly, we explore the added benefits of data augmentation, on top of BoW and DA, for cross-language SER.

The remainder of this paper is organized as follows: Section 2 describes related literature in cross-language SER, multilingual training, and data augmentation for SER, DA, and domain generalization. Section 3 describes the proposed method along with the materials and methods used, while Section 4 presents the experimental setup. Section 5 presents the experimental results and discusses them, and finally, Section 6 draws the conclusions.

## 2. Related Work

In this section, we describe related work dealing with cross-language, multilingual training and the data augmentation, domain adaptation, and generalization aspects of speech emotion recognition.

### 2.1. Cross-Language SER

The primary goal of an SER system is to detect emotions within a speech signal. As available datasets are typically recorded in one language, the majority of existing systems have reported monolingual results. Under cross-corpus conditions, however, especially with systems trained on one language and tested on another, system performance can decay drastically [1,2,3,4]. The work in [6], for example, proposed the use of feature and speaker normalization to remove language effects from the SER system, and experiments across six languages showed the importance of multilingual training paradigms. The work in [23], on the other hand, showed the importance of feature selection for cross-language SER. The experiments in [24] showed that gender- and language-specific models could be used to improve cross-language SER accuracy between Mandarin and other western languages. They reported higher performance in cross-language families compared to within-language families, suggesting some universal cues of emotional expression, regardless of language. Overall, cross-language SER accuracy was shown to be higher for the arousal dimension relative to valence [1,23].

In the work described in [25], subspace-alignment-based domain adaptation schemes were used to map language-specific SER models to unseen languages. Chiou and Chen, in turn, explored data normalization using histograms for improved cross-language SER [9]. Furthermore, the authors in [26] used deep belief networks to learn generalized features across different languages and showed improved cross-corpus SER performance. Ning et al., in turn, showed that universal feature representations could be achieved with bidirectional long short-term memory (Bi-LSTM) neural networks with shared hidden layers trained on English and Mandarin speech [27]. Several other works (e.g., [28,29,30]) have explored the use of end-to-end deep neural networks trained on multilingual data for improved cross-language SER. Multilingual SER can also be seen as a form of data augmentation; thus, the next section focuses on multilingual training and data augmentation for cross-lingual SER.

### 2.2. Multilingual Training and Data Augmentation for SER

Hesam et al. showed the benefits of using language identification coupled with multi-language SER models [10] for cross-language SER. In fact, multi-language training (i.e., training models with data from more than one language) has been shown to attain reliable SER predictions for unseen languages [31,32,33,34]. Schuller et al. showed the effect of selecting only the most prototypical examples when training cross-dataset SER systems [35] and later showed the importance of fusing the outputs of different deep learning systems [31]. More recently, convolutional neural networks (CNN) with attention have been proposed for cross-language SER [2,7], where multi-language training was shown to improve cross-language SER performance. In [2,36], data augmentation was also shown to improve cross-language SER accuracy.

### 2.3. Domain Adaptation for SER

Domain adaptation aims to improve the generalization capacity of models by adapting the domain shift of the source or target data, thus minimizing the differences in the feature space between both domains. Zhang et al. tackled cross-language SER by separately normalizing the features of each speech corpus [33]. Hassan et al., in turn, employed kernel mean matching to increase the weight of the training data to match that of the test data distribution [11]. Zong et al. used least square regression to remove the projected mean and covariance differences between the source data and unlabeled target samples while learning the regression coefficient matrix [37], thus proposing a domain-adaptive least-squares regression model for cross-corpus SER. Song et al. proposed a novel DA method based on dimensionality reduction to create a similar feature space for both source and target domains [38]. Abdelwahab et al., in turn, explored a model-based DA method in which supervised adaptation of a support vector machine (SVM) classifier was performed via access to small amounts of target domain data [39].

Furthermore, the work in [8] proposed kernel canonical correlation analysis (KCCA) on principal component subspaces for DA. They first projected the source and target to the feature space using PCA applied on the combined source and target domains. Then, they used KCCA to maximize the correlation between both. Song et al., in turn, proposed a non-negative matrix factorization-based DA for cross-language SER [12]. More specifically, the authors proposed an algorithm that aimed to represent a matrix formed by data from both the source and target domains as two non-negative matrices whose product was an approximation of the original matrix. In order to ensure that the differences in the feature distributions of the two corpora were minimized, they regularized this factorization by the maximum mean discrepancy. Moreover, Abdelwahab and Busso [40] proposed a semi-supervised approach by creating ensemble classifiers. In this method, each classifier focuses on a different feature space, thus learning the discriminant features for the target domain.

### 2.4. Domain Generalization for SER

Domain generalization differs from domain adaptation, in which the training and test domains are mapped to a common space where the feature representation is more robust to the variations between the domains. In [41], a sparse autoencoder method was used for feature transfer learning for SER. A common emotion-specific mapping rule is first learned from a small set of labeled data in a target domain. Then, this rule is applied to emotion-specific data in a different domain. Deng et al. [42], in turn, used autoencoders to find a common feature representation between the source and target domains by minimizing the reconstruction error on both domains. Later, Mao et al. [43] proposed learning a shared feature representation by sharing the class priors across domains. The work in [44] proposed Universum autoencoders, where the Universum loss is added to the reconstruction loss of an auto-encoder to reduce the reconstruction and classification error on both the source and target domains. Deng et al. also presented a denoising autoencoder-based approach for cross-language SER [45,46]. In fact, several variations of autoencoders have been investigated for cross-language SER, including variational autoencoders (VAE) [47], adversarial autoencoders (AAE) [48], and adversarial variational Bayes (AVB) [48].

## 3. Proposed Method

**Figure 1:**
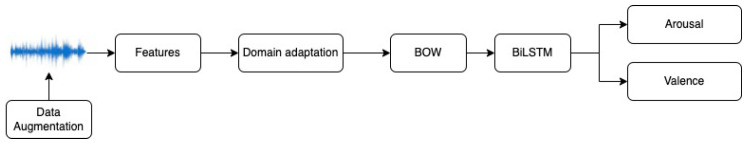
Block diagram of the cross-language SER systems combining BoW and domain adaptation.

This section describes the proposed method based on the combination of bag-of-word (BoW) signal methodology and domain adaptation for cross-language SER. Figure 1 depicts a block diagram of the two methods explored herein, where the BoW feature extraction methodology is explored before or after domain adaptation. More details about each individual block are described next.

### 3.1. Speech Feature Extraction

SER systems rely on different speech feature representations. In previous AVEC Challenges, hand-crafted features have been compared against feature representations obtained directly from end-to-end deep neural networks. It has been observed that hand-crafted features still outperform deep-spectrum-based features, for example [17]. This is likely due to the fact that existing emotion-labeled datasets are fairly small compared to other domains, such as speech recognition, in which large amounts of data are available to allow for accurate feature representations to be obtained directly from the model. As the datasets used herein are fairly small, we employ two popular feature representations, namely eGeMAPS and modulation spectral features (MSF). Here, the openSMILE toolkit is used to extract the extended Geneva Minimalistic Acoustic Parameter Set (eGeMAPS) [49], which has been widely used in various recent SER challenges (e.g., [18,50,51]). In particular, eGeMAPS contains a set of 88 acoustic parameters relating to pitch, loudness, unvoiced segments, temporal dynamics, and cepstral features. In our pilot experiments, however, we have found that a subset of 23 eGeMAPS features performed better when combined with the bag-of-words methodology (as detailed in the next section), so in our experiments described herein, a subset of 23 features are used. In particular, these 23 LLDs also include loudness, voicing-related features, pitch, cepstral features, and temporal dynamics. Modulation spectral features are also explored as they capture spectral and temporal information from the speech signal and have been shown to not only convey emotional information but also provide some robustness against environmental factors [52,53]. Modulation spectral features were extracted using a window size of 256 ms and a frame step of 40 ms, following the steps described in [53]. We fuse these two feature sets into a final feature vector of dimension 246, of which 23 correspond to eGeMAPS and 223 to MSF features.

### 3.2. Bag-of-Words Methodology

The bag-of-words (BoW) methodology was initially proposed for natural-language-processing applications [54]. However, this approach has also captured the attention of applications where low-level descriptors (LLDs; i.e., features at a short time scale) are employed, yielding performance improvements for speech, audio, video, and other modalities [55]. In audio processing, BoW has been utilized with the term bag-of-audio-words, where LLDs are extracted from the audio signal and then codebook quantized [56]. Generally, statistical functionals such as mean, standard deviation, minimum, and maximum have been widely employed to represent frame-level features as utterance-level features. BoW is an alternative representation that aggregates frame-level features into utterance-level ones using different clustering methods. BoW has been utilized for music information retrieval [57] and, more recently, for SER [58].

**Figure 2:**
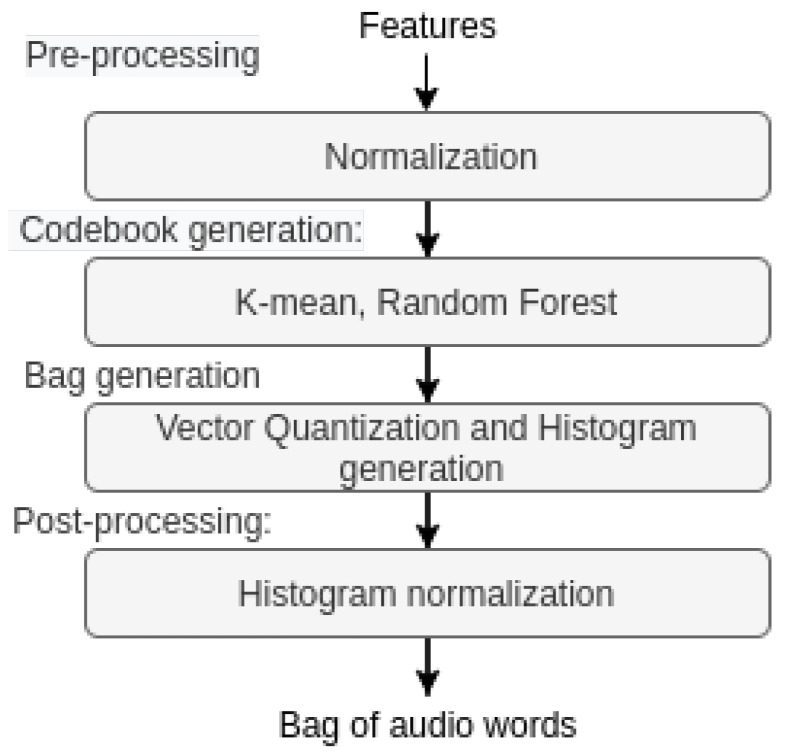
Steps for bag of audio word generation.

Figure 2 depicts the steps involved in BoW feature processing. In this method, we first pre-process the LLDs using different normalization methods, such as min-max scaling, linear scaling, and z-score. These normalized features are then input to the codebook generation algorithm. There are different methods for codebook generation, such as random sampling or K-means clustering. The histogram represents the frequency of occurrence of each feature or word. The histograms are generated from the frequencies of each numeric value. As a final post-processing step, the resulting histogram is normalized using term frequency (TF) weighting and inverse document frequency (IDF) weighting, and the resultant vector is then fed to a regression model [58]. Here, we explore the usefulness of applying the BoW methodology after domain adaptation for cross-language SER. More details about the BoW procedure can be found in [19,58]. The code for BoW generation can be found at https://github.com/shrutikshirsagar/cross-language-SER (accessed on 19 August 2022). In particular, we employed Z-score standardization and random sampling for codebook generation. The random-sampling-based codebook generation is much faster than the k-mean-clustering-based algorithm.

### 3.3. Domain Adaptation/Generalization

Here, two domain adaptation methods are explored, and one domain generalization method is proposed. More details are provided in the sub-sections to follow.

#### 3.3.1. Subspace Alignment-Based Domain Adaptation

SA-DA aims to find a domain-invariant feature space by learning a mapping function that aligns the source subspace with the target one [59]. SA-DA linearly aligns the source domain to the target domain in a reduced-dimension PCA subspace. In this method, we first create subspaces for both the source and target domains and then learn a linear mapping that aligns the source subspace with the target subspace. This allows comparisons of the source domain data directly with the target domain data and allows one to build classifiers on source data and apply them to the target domain. With SA-DA source data *S*, target data *T*, source labels LS, and subspace dimension *D*, source data S and target data T are first projected into the D dimension via the following equations:

(1) $$X_{S}=PCA(S,D),$$

(2) $$X_{T}=PCA(T,D),$$

where $X_{S}$ and $X_{T}$ represent the projected source and target data, respectively. Moreover,

(3) $$X_{A}=X_{S}X_{S}^{\prime }X_{T},$$

where $X_{S}^{\prime }$ represents the transpose of the projected source data. Lastly,

(4) $$S_{A}=SX_{A},$$

(5) $$T_{T}=TX_{T}.$$

In our experiment, the resultant feature embedding of source $S_{A}$ and target domain $T_{T}$ can be either input to a deep-learning-based regression model for arousal and valence prediction (in Method 1) or further processed via the BoW methodology prior to emotion recognition (Method 2).

#### 3.3.2. Correlation Alignment

We also explored the unsupervised CORAL algorithm [22], which matches the first- and second-order statistics of the source and target data. Let the source domain correspond to the training data language, and the target domain, the test set language; the CORAL algorithm first calculates the statistics of the target domain and then subtracts the covariance of the target domain from the source domain by whitening and recoloring the source domain.

More specifically, the source domain feature matrix $D_{source}$ is first whitened and given by $D_{source}^{w}$, that is,

(6) $$D_{source}^{w}=D_{source}*C_{source}^{-\frac{1}{2}},$$

where ∗ represents matrix multiplication.

The matrix is recolored ($D_{source}^{adapted}$) by the following:

(7) $$D_{source}^{adapted}=D_{source}^{w}*C_{target}^{\frac{1}{2}},$$

where $C_{source}$ and $C_{target}$ are given by

(8) $$C_{source}=\Sigma_{source}+I,$$

(9) $$C_{target}=\Sigma_{target}+I.$$

Here, *I* corresponds to the identity matrix, and $\Sigma_{source}$ and $\Sigma_{target}$ to the covariance matrices of the source and target domains, respectively.

#### 3.3.3. Domain Generalization with CORAL

**Figure 3:**
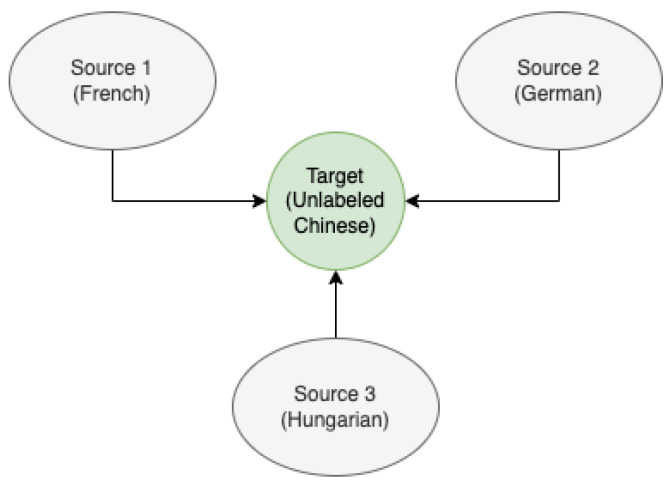
Proposed N-CORAL-based domain generalization strategy for cross-language SER.

We propose a variant of the described approach where a third language is used to adapt both the training and test domains. Figure 3 depicts this domain generalization method, which we term N-CORAL. In our experiments, we utilize a Chinese language dataset as the target domain and adapt the training and test data of three different languages, namely German, French, and Hungarian, to this common domain before training an SER classifier. The main advantage of the proposed method is that we do not need access to the test data, as in previous methods. The same whitening and recoloring equations from (6)–(9) are used, but now for a common language.

## 4. Experimental Setup

In this section, we describe the databases used, proposed regression model architectures, benchmark systems, and figure-of-merit used to gauge system performance.

### 4.1. Databases

For emotion prediction, we employed four datasets in four different languages. The first corresponds to the Remote Collaborative and Affective Interactions (RECOLA) database [60]. This database was used during the 2016 audio-visual emotion challenge (AVEC) [51] and is in the French language. Based on spontaneous and naturalistic interactions collected from a collaborative task, six annotators measured emotion continuously using a time-continuous scale for two emotion primitives, namely arousal and valence. Even though all subjects were fluent French speakers, they came from different nationalities (French, Italian, and German); thus, the database provides some diversity in the expression of emotion. In addition, the total number of speakers in the RECOLA dataset was 27, out of which 16 were females, and 11 were males. Detailed participant statistics are available in [60]. The subjective labels were originally available with a frame rate of 40 ms. We aggregated five consecutive frames to generate a frame rate of 200ms for analysis via averaging. The RECOLA database is partitioned into three disjoint sets: training, development, and test, each containing 5-minute-long speech files from nine speakers.

The second and third datasets correspond to the German and Hungarian language subsets of the Sentiment Analysis in the Wild (SEWA) database. This database was used in the AVEC 2018 [17] and AVEC 2019 [18] challenges. Subjects (in pairs of friends and relatives) were recorded through a dedicated video chat platform, using their own standard web cameras and microphones while they discussed an advertisement they had watched. Detailed participant demographics for both datasets are available in [61]. The duration of the recordings in the dataset range from 40 seconds to 3 minutes for each file. Both datasets are divided into 34 files for training, 14 in the development set, and 16 for testing. In our experiments, we only used the training and development parts, as the labels were not available for the test set. The SEWA dataset has valence and arousal labels available, with a frame rate of 100 ms; thus, we aggregated two consecutive frames via averaging to remain consistent with the frame durations used with the RECOLA dataset. Both the RECOLA and SEWA datasets were recorded with a sampling rate of 44.1 kHz. Further details about the dataset can be found in [61].

The fourth dataset corresponds to the Chinese language subset of the SEWA project. The audio recordings’ sample rate was 44.1 kHz, and the total data duration is 3:17:52 hours. There were audio samples from 36 male and 34 female participants. A total of 70 audio files without labels were made available through the AVEC 2019 [18] challenge. Detailed participant demographics for this dataset are available in [61]. In our experiment, we specifically used this unlabeled dataset for the proposed N-CORAL domain generalization method described in Section 3.3.3. We downsample all audio files to 16 kHz for further processing.

Next, we used the recorded noise dataset AURORA [62] for data augmentation purposes to further corrupt the SEWA and RECOLA datasets. More specifically, two noise types, multi-talker babble and noise recorded inside a commercial airplane, were used to corrupt data. We further added noise at five different signal-to-noise levels (SNRs): 0 dB, 5 dB, 10 dB, 15 dB, and 20 dB. Moreover, three recorded room impulse responses taken from [63] were used and convolved with the speech files to simulate room reverberation at different reverberation times, namely T60=0.25, 0.48, and 0.8 seconds.

### 4.2. Regression Model

Recurrent neural networks (RNN) are a family of neural networks and are extremely useful for handling sequential data as their output corresponds to a specific combination of current and past inputs. However, due to the nature of the long sequence, they usually suffer from vanishing/exploding gradient problem. In order to solve this problem, LSTM-RNNs were later proposed [64]. A bidirectional LSTM, or BiLSTM, is a sequence-processing model that consists of two LSTMs, one taking the input in a forward direction and the other in a backward direction. BiLSTMs effectively increase the amount of information available to the network, improving the content available to the algorithm. BiLSTMs and LSTMs have been widely used in speech applications (e.g., [17,27,65,66]).

We employed the benchmark architecture from the AVEC 2018 [17] and AVEC 2019 challenges described in [18]. We used two-layer BiLSTM with hidden layers of sizes 64 and 32, respectively. A whole sequence was used for training, and the experiment lasted 1000 epochs. A concordance correlation coefficient (CCC)-based loss function (see Section 4.5) was used for training inspired by the AVEC 2018 and 2019 benchmark systems. We used TensorFlow with KERAS as a backend. The implemented model for experimental validation can be found at https://github.com/shrutikshirsagar/cross-language-SER (accessed on 19 August 2022). Finally, for the hyper-parameter search, we used the validation set. We experimented with three widely used optimizers, RmsProp, Adam, and SGD, with three different learning rates (0.01, 0.001, 0.0001) and varying dropout (0.1–0.5 in 0.1 increments).

### 4.3. Data Augmentation

As mentioned previously, data augmentation has been widely used to improve model generalizability against training/test mismatch. In fact, recent results have shown data augmentation to provide some robustness to language mismatch [19]. Here, we aim to explore the additional benefits that data augmentation can bring for cross-lingual SER when combined with other strategies, such as domain adaptation and BoW. In particular, we augment the unprocessed speech training datasets with (i) two different noise types (babble and airport) at five different signal-to-noise ratios (SNR = 0, 5, 10, 15, and 20 dB); (ii) three reverberation levels to simulate a small, medium, and large-sized room (RT60 = 0.25, 0.48, and 0.80 s); and (iii) 12 noise-plus-reverberation conditions (2 noises × 3 SNR levels (0, 10, and 20 dB) × 2 RT60 values (0.25, 0.8 s)).

### 4.4. Benchmark Systems

Several benchmarks are used to gauge the benefits achieved with the proposed SER system. In particular, SA-DA alone [59] and CORAL-DA alone are used as benchmarks [22], as well as BoW alone, and no processing. We also used the AVEC 2019 challenge baseline [17] as an additional benchmark, as it relied on a bag-of-words methodology but over eGeMAPS features alone, together with an LSTM regressor. Furthermore, in order to demonstrate the usefulness of the proposed approach, we compared it with several other methods, including a transfer learning approach based on principal component analysis (PCA), as in [67]; a canonical-correlation-analysis-based method (KCCA), as in [8]; and structural correspondence learning (SCL), as in [68]. Lastly, we use only data augmentation as a benchmark system.

### 4.5. Figure-of-Merit, Testing setUp, and Experimental Aims

The performance measure used here is the typical metric used within SER tasks, that is, the *concordance correlation coefficient* (CCC). This figure-of-merit combines Pearson’s correlation coefficient ρ with the square difference between the mean of the two compared time series and is given as follows (Equation (10)):

(10) $$\mathrm{CCC}=\frac{2\rho \sigma_{x}\sigma_{y}}{\sigma_{x}^{2}+\sigma_{y}^{2}+{\left(\mu_{x}-\mu_{y}\right)}^{2}},$$

where μ and σ stand for the first and second order statistics of times series *x* and *y*, which correspond to the emotion predictions and their corresponding subjective ratings, respectively.

For the experimental setup, we have employed only the labeled training and validation partitions of the AVEC challenge datasets. More specifically, we used the training data as our training data, and further divided this training data (80%/20%) for hyper-parameter tuning of the Bi-LSTM models. In addition, our test set is the challenge validation set. In the end, once we found appropriate parameters, including optimizer, learning rate, and drop-out, for the model’s final training, we joined our training and validation sets (which were earlier divided as 80%/20%). We also showed the significance of the obtained results using a z-score test between the CCCs. In particular, we used a 95% level (p<0.05) against the AVEC 2019 benchmark system.

We start the experiments with an ablation study aimed at measuring the upper bound achieved per language using monolingual models where the same language is used for training and testing. Next, we examine the impact of multilingual training, where multiple languages are combined during training. Next, we explore the impact of including the bag-of-words methodology, domain adaptation schemes, and data augmentation, both individually and combined. Lastly, we experiment with the proposed N-CORAL method.

## 5. Experimental Results and Discussion

In this section, we present the experimental results and then discuss our findings in light of the existing literature.

### 5.1. Ablation Study

As an ablation study, we explored monolingual and multilingual training experiments to obtain “upper bounds” on what could be achieved for the tested languages and datasets without any domain adaptation strategies in place. Monolingual refers to experiments where the language of the test samples are the same as those used during training. Multilingual, in turn, combines multiple languages during training and tests them individually with either matched or unseen languages. Table 1 presents the ablation study results for several experiments, including three monolingual (training/testing in German, Hungarian, and French), three multilingual (training with German/Hungarian/French and testing with each language individually), three unseen multilingual (training with two languages and testing with the third unseen), and lastly, the three unseen multilingual conditions, but with data augmentation during training. For all experiments, BoW features and a BiLSTM regressor were used.

For these experiments, 34 audio files for training and 14 audio files for testing were used from the SEWA-German and SEWA-Hungarian datasets, whereas nine audio files were used for training, and nine audio files were used for testing from the RECOLA-French dataset. As can be seen from Table 1, valence estimation is more challenging compared to arousal, corroborating findings in [1,23]. Moreover, with the exception of the Hungarian language, multi-language training did not help improve accuracy over the monolingual settings. Having the test language present during training was shown to be important. Lastly, in the case where the test language was unseen, data augmentation was shown to be important.

### 5.2. Proposed System

Table 2 and Table 3 show the cross-language results obtained under the different conditions explored herein for arousal and valence prediction, respectively. Cross-language results achieved with different benchmarks (see Section 4.4) and the proposed systems are reported. In the tables, a column labeled ‘G-H’ means that German and Hungarian languages were used for training and testing, respectively. As previously, for training, we used the 34 (training) audio files from the SEWA-G and SEWA-H datasets and 9 French-language audio files from the RECOLA dataset. For testing, we used 14 audio files from the SEWA-G and SEWA-H test sets and 9 from RECOLA. Significant improvements relative to the AVEC 2019 benchmark are reported with an asterisk. All results rely on the BiLSTM model.

**Figure 4:**
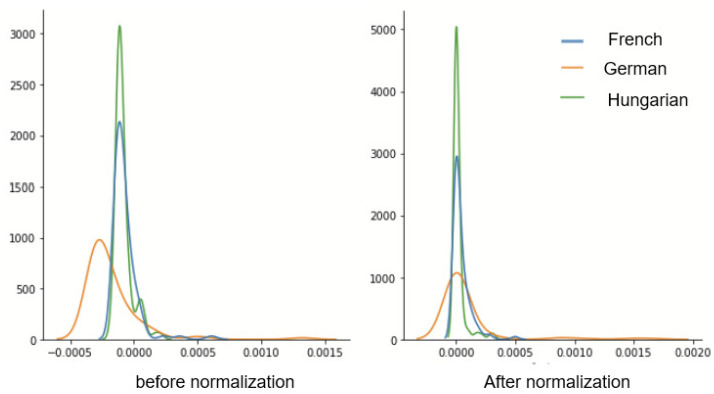
Illustration of the effects of CORAL on the distribution of one MSF feature for French, German, and Hungarian languages. Plots on the left are before normalization, and those on the right are after normalization.

As can be seen, for arousal, all of the proposed methods significantly outperformed the AVEC 2019 benchmark system on average. This benchmark is based on BoW applied to eGeMAPS features combined with an LSTM regressor. Interestingly, the other benchmark based on applying only BoW on the combined eGeMAPS and MSF features, together with a BiLSTM, was also shown to significantly improve arousal prediction accuracy over the AVEC 2019 benchmark, thus highlighting the importance of the MSF features for the task at hand. Moreover, comparing the two proposed domain adaptation methods (SA-DA + BoW and CORAL + BoW), on average, the CORAL-based method achieved the highest CCC. The left- and right-side plots in Figure 4 depict the histograms of one MSF feature for the French, German, and Hungarian languages before and after CORAL normalization, respectively. As can be seen, CORAL reduces the shift between the distributions across languages, resulting in improved cross-language accuracy.

Overall, the proposed N-CORAL method achieved the highest CCC values of all tested methods, also outperforming several multilingual settings, as shown in Table 1. This was followed closely by the proposed CORAL + BoW setting. Data augmentation, in turn, helped improve performance for half of the cross-language tasks, but on average, it did not provide any significant advantage for the N-CORAL setting. Moreover, it can be seen that in the conditions involving the SEWA German and SEWA Hungarian cross-language tasks, the highest CCC values across all tested cross-language tasks was achieved, especially with the N-CORAL + BoW method. These findings suggest that such a proposed scheme can be useful for cross-language normalization but not necessarily for cross-corpus normalization, where other nuance factors may be present. For cross-corpus and cross-language robustness, N-CORAL combined with BoW and data augmentation showed the most significant gains, combining the benefits of the N-CORAL + BoW method for cross-language robustness and the benefits of data augmentation for cross-corpus nuance factors.

Moreover, comparing the results from the tables, it can be seen that valence prediction is a more challenging task compared to arousal prediction, corroborating previous findings [1,31]. Notwithstanding, the proposed methods were shown to reduce this gap across many of the cross-language tasks. Overall, all of the proposed methods achieved CCC values significantly better than most benchmarks. Similar to the arousal prediction case, the proposed N-CORAL and CORAL + BoW settings achieved the highest average results. For valence prediction, data augmentation only helped for two of the six tested cases.

**Figure 5:**
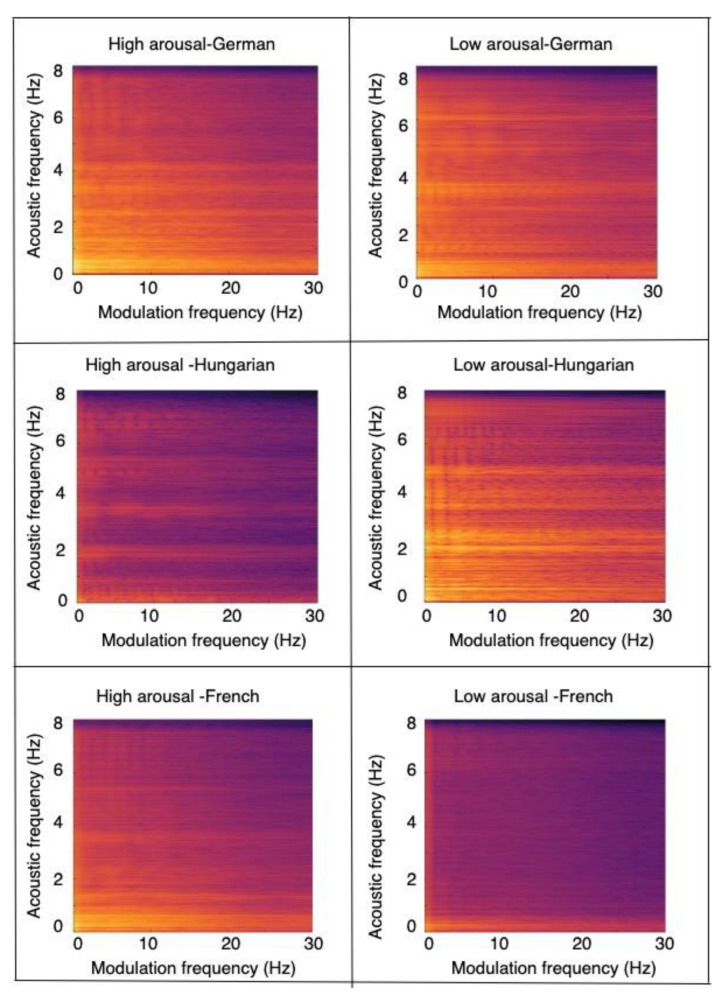
Average modulation spectrogram for German (top), Hungarian (middle), and French (bottom) language for high- (left) and low- (right) arousal conditions.

**Figure 6:**
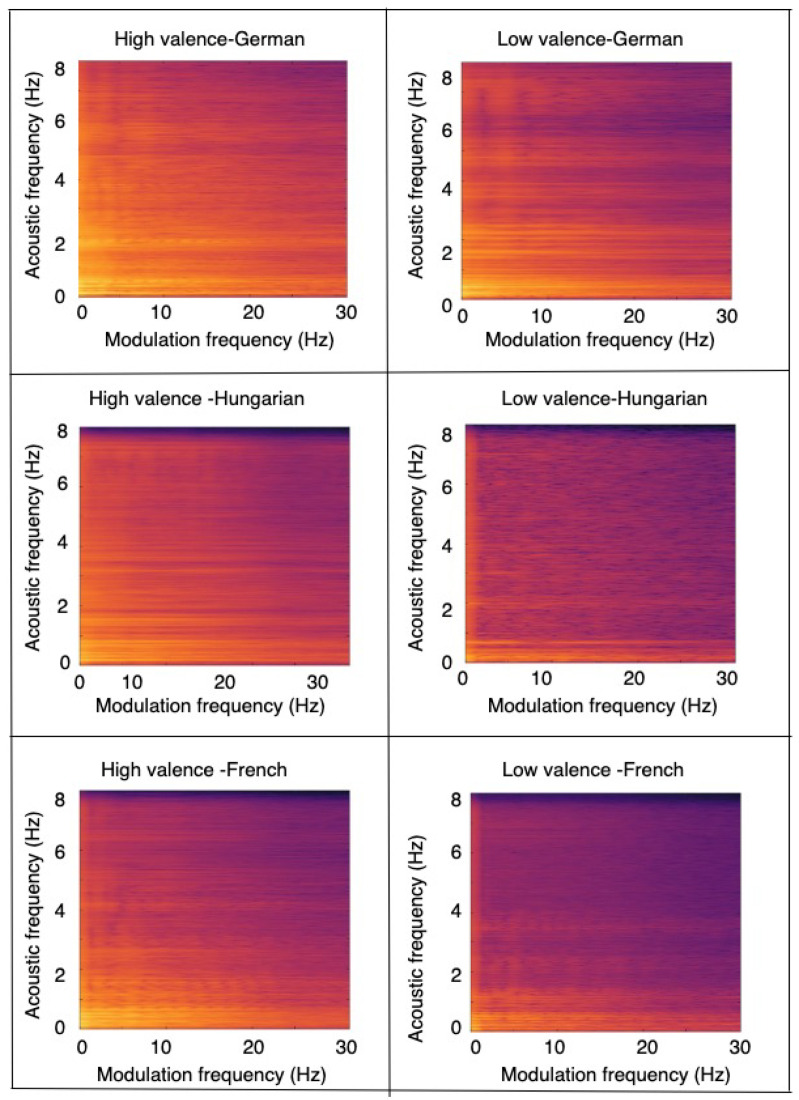
Average modulation spectrogram for German (top), Hungarian (middle), and French (bottom) language for high-(left) and low-(right) valence conditions.

To better understand some of these findings, Figure 5 depicts a snapshot of the average modulation spectrogram across multiple speakers for three different languages for both high- (left) and low- (right) arousal conditions. As can be seen, differences across languages can be seen for both high- and low-arousal cases, motivating the need for cross-language strategies. Apart from the language differences, differences can also be seen between the high- and low-arousal conditions. Figure 6, on the other hand, shows modulation spectrograms for high- (left) and low- (right) valence conditions across the three languages. As can be seen, the differences are more subtle, suggesting a more complex classification task.

Furthermore, it can be observed that that while utilizing only data augmentation to compensate for cross-language issues can provide some improvements relative to doing nothing, the gains are typically substantially lower than applying other domain adaptation strategies. This can be somewhat expected as the augmentation strategies comprised adding noisy versions of the same language.

Lastly, while the proposed N-CORAL method was shown to achieve the best performance across several valence and arousal prediction tests, further improvements may be achieved if other target languages are used. Here, the Chinese unlabeled data from the SEWA dataset was used as it was available together with other emotion-labeled subsets. As Chinese belongs to a different family of languages than the other source languages experimented with here, future work may explore the use of different target languages belonging to the same family as the source languages to see if further gains can be achieved.

## 6. Conclusions

In this paper, we explored combined use of the bag-of-words methodology, domain adaptation, and data augmentation as strategies to counter the detrimental effects of cross-language (and cross-corpus) speech emotion recognition. A new method termed N-CORAL was also proposed in which all languages are mapped to a common distribution (in our case, a Chinese language model). Experiments with German, French, and Hungarian languages show the benefits of the proposed N-CORAL method, combined with data augmentation and BoW for cross-language SER. 

## Figures and Tables

**Table 1:** Ablation study results for monolingual, multilingual with matched test language, and multilingual with unseen test language experiments, without and with (‘+Aug’) data augmentation.

Train	Test	Arousal	Valence
German	German	0.450	0.363
AVEC 2019	German	0.434	0.455
Multi-matched	German	0.399	0.318
Multi-unseen	German	0.067	0.150
Multi-unseen + Aug	German	0.179	0.187
Hungarian	Hungarian	0.123	0.145
AVEC 2019	Hungarian	0.291	0.135
Multi-matched	Hungarian	0.263	0.154
Multi-unseen	Hungarian	0.147	0.037
Multi-unseen + Aug	Hungarian	0.241	0.240
French	French	0.772	0.418
AVEC 2019	French	0.323	0.144
Multi-matched	French	0.538	0.186
Multi-unseen	French	0.046	0.045
Multi-unseen + Aug	French	0.157	0.164

**Table 2:** Performance comparison of arousal estimation with different explored schemes in terms of CCC. Bi-LSTM regression was used for all methods. Highest values are indicated in bold, and significantly better results relative to benchmark are highlighted by an asterisk.

Systems	Settings	Arousal						
		G-H	G-F	F-H	F-G	H-F	H-G	Avg
Benchmark	AVEC 2019	0.160	0.143	0.134	0.312	0.021	0.698	0.244
	No processing	0.118	0.128	0.144	0.237	0.045	0.711 *	0.230
	BoW only	0.179 *	0.131	0.155 *	0.320	0.115 *	0.749 *	0.274 *
	PCA	0.130	0.146	0.097	0.125	0.028	0.717 *	0.207
	KCCA	0.180 *	0.228 *	0.123	0.082	0.180 *	0.674	0.244
	SCL	0.124	0.165*	0.141	0.198	0.037	0.766 *	0.238
	SA-DA	0.140	0.151	0.122	0.148	0.195 *	0.762 *	0.253
	CORAL	0.125	0.236 *	0.124	0.161	0.119 *	0.729 *	0.249
	Data augmentation	0.201 *	0.129	0.220 *	0.119	0.150 *	0.447	0.211
Proposed	SA-DA + BoW	0.193 *	0.154	0.188 *	0.248	0.109 *	0.765 *	0.276 *
	CORAL + BoW	0.268*	0.167 *	0.138	0.433 *	0.138 *	0.739*	0.313*
	N-CORAL	0.207*	0.127	0.167 *	0.278	0.148 *	0.733 *	0.276 *
	N-CORAL + BoW	0.282 *	0.126	0.175 *	0.464 *	0.124 *	0.787*	0.326 *
	N-CORAL + BoW + Aug	0.193 *	0.189 *	0.241 *	**0.369**	0.156 *	0.480	0.271 *

**Table 3:** Performance comparison of arousal estimation with different explored schemes in terms of CCC. Bi-LSTM regression was used for all methods. Highest values are indicated in bold, and significantly better results relative to benchmark are highlighted by an asterisk.

Systems	Settings	Valence						
		G-H	G-F	F-H	F-G	H-F	H-G	Avg
Benchmark	AVEC 2019	0.046	0.112	0.200	0.073	0.090	0.671	0.198
	No processing	0.014	0.104	0.204	0.130 *	0.109 *	0.719 *	0.213 *
	BoW only	0.074 *	0.133 *	0.260 *	0.153 *	0.141 *	0.745 *	0.251 *
	PCA	0.031	0.160 *	0.137	0.104 *	0.048	0.712 *	0.198
	KCCA	0.069 *	0.129	0.165	0.069	0.057	0.641	0.188
	SCL	0.024	0.157 *	0.169	0.092 *	0.071	0.771 *	0.214 *
	SA-DA	0.033	0.126	0.128	0.117 *	0.117 *	0.782 *	0.217 *
	CORAL	0.065 *	0.168 *	0.315 *	0.113 *	0.09	0.726 *	0.246 *
	Data augmentation	0.107 *	0.141 *	0.214	0.075	0.154 *	0.32	0.165
Proposed	SA-DA + BoW	0.094 *	0.139 *	0.114	0.130 *	0.158 *	0.778 *	0.235 *
	CORAL + BoW	0.128 *	0.200 *	0.371 *	0.202 *	0.123 *	0.681 *	0.284 *
	N-CORAL	0.062 *	0.125	0.143	0.131 *	0.078	0.752 *	0.215 *
	N-CORAL + BoW	0.141 *	0.169 *	0.217 *	0.310 *	0.051	0.799 *	0.281 *
	N-CORAL + BoW + Aug	0.129 *	0.131 *	0.352 *	0.247 *	0.169 *	0.473	0.267 *

## Data Availability

Not applicable.
